# Long-Term Soy Protein Isolate Consumption Reduces Liver Steatosis Through Changes in Global Transcriptomics in Obese Zucker Rats

**DOI:** 10.3389/fnut.2020.607970

**Published:** 2020-12-11

**Authors:** Melisa Kozaczek, Walter Bottje, Byungwhi Kong, Sami Dridi, Diyana Albataineh, Kentu Lassiter, Reza Hakkak

**Affiliations:** ^1^Department of Dietetics and Nutrition, University of Arkansas for Medical Sciences, Little Rock, AR, United States; ^2^Department of Poultry Science & The Center of Excellence for Poultry Science, University of Arkansas, Fayetteville, AR, United States; ^3^Department of Pediatrics, University of Arkansas for Medical Sciences, Little Rock, AR, United States; ^4^Arkansas Children's Research Institute, Little Rock, AR, United States

**Keywords:** liver steatosis, zucker rat, soy protein isolate, liver transcriptomics, RNAseq, obesity

## Abstract

To determine how soy protein isolate (SPI) ameliorated liver steatosis in male obese Zucker rats, we conducted global transcriptomic expression (RNAseq) analysis on liver samples of male rats fed either the SPI or a control casein (CAS)-based diet (n = 8 per group) for 16 weeks. Liver transcriptomics were analyzed using an Ilumina HiSeq system with 2 × 100 base pair paired-end reads method. Bioinformatics was conducted using Ingenuity Pathway Analysis (IPA) software (Qiagen, CA) with *P* < 0.05 and 1.3-fold differential expression cutoff values. Regression analysis between RNAseq data and targeted mRNA expression analysis of 12 top differentially expressed genes (from the IPA program) using quantitative PCR (qPCR) revealed a significant regression analysis (*r*^2^ = 0.69, *P* = 0.0008). In addition, all qPCR values had qualitatively similar direction of up- or down-regulation compared to the RNAseq transcriptomic data. Diseases and function analyses that were based on differentially expressed target molecules in the dataset predicted that lipid metabolism would be enhanced whereas inflammation was predicted to be inhibited in SPI-fed compared to CAS-fed rats at 16 weeks. Combining upstream regulator and regulator effects functions in IPA facilitates the prediction of upstream regulators (e.g., transcription regulators) that could play important roles in attenuating or promoting liver steatosis due to SPI or CAS diets. Upstream regulators that were predicted to be activated (from expression of down-stream targets) linked to increased conversion of lipid and transport of lipid in SPI-fed rats included hepatocyte nuclear factor 4 alpha (HNF4A) and aryl hydrocarbon receptor (AHR). Upstream regulators that were predicted to be activated in CAS-fed rats linked to activation of phagocytosis and neutrophil chemotaxis included colony stimulating factor 2 and tumor necrosis factor. The results provide clear indication that long-term SPI-fed rats exhibited diminished inflammatory response and increased lipid transport in liver compared to CAS-fed rats that likely would contribute to reduced liver steatosis in this obese Zucker rat model.

## Introduction

Excess fat accumulation in the liver, liver steatosis, a condition strongly linked to obesity, can be divided in two major categories: Alcoholic Fatty Liver Disease and Non-Alcoholic Fatty Liver Disease (NAFLD). Alcoholic liver disease occurs due to alcohol abuse over extended period of time (1), whereas NAFLD is described as an abnormal amount of lipid present in liver cells that is not due to alcohol intake (2). In the last decade, NAFLD has gained more attention due to its high connection with other disorders, such as an increase in the lipids in the circulation, excess body weight, insulin resistance, and inflammation, diabetes type II, and vascular disorders (2, 3) that when present at the same time contribute to a larger disorder called Metabolic Syndrome. Metabolic syndrome has its origin in obesity with hereditary components and sedentary behavior (4).

If left untreated, the mild steatosis in NAFLD can lead to non-alcoholic steatohepatitis (NASH), and finally to irreversible damage to the liver due to cirrhosis. The development of NAFLD occurs in two stages; (Stage 1) Insulin resistance develops that is accompanied by lipid (triglyceride) accumulation in the liver, and (Stage 2) Mitochondrial dysfunction that stimulates mitochondrial reactive oxygen species production leading to oxidative stress, inflammation, and hepatic fibrosis (5, 6). A revision of the progression of pathogenesis has been proposed in which NAFLD is diagnosed in patients with elevated levels of circulating liver enzymes and by imaging devoid of other causes of liver disease. However, a definitive diagnosis can only be made by liver biopsy (7, 8). NAFLD is likely the most common form of chronic liver disease in adults in the US, Asia, Australia, and Europe that affects 10–35% of the worldwide population (9) and there is concern of NAFLD as a significant form of liver disease in pediatric populations (10, 11).

Feeding soy protein isolate (SPI) specifically targeted and halted the development of liver steatosis in obese Zucker rats (males and females) compared to ones fed a casein (CAS)-based diet (12). However, the precise mechanisms that lead to SPI-mediated attenuating liver steatosis is not completely understood. To better understand fundamental mechanisms by which SPI attenuated NAFLD, we conducted global transcriptomic (RNAseq) analysis on liver tissue obtained from obese rats fed the SPI- and CAS-based diets for 8 weeks (13). The results of this study indicated gene expression that would promote anti-inflammatory activities in SPI-fed rats and inflammatory activities in CAS-fed rats, which is consistent with the observation of reduced liver steatosis in SPI-fed rats (12). In the present study, we report on global expression analysis of liver obtained after long-term soy feeding (16 weeks of feeding CAS- and SPI-based diets) to obese rats to intensify efforts to reveal basic mechanisms of liver steatosis development and amelioration of liver steatosis afforded to obese rats consuming the SPI-based diet.

## Materials and Methods

### Experimental Design

Animal protocols were approved by the Institutional Animal Care and Use Committee at the University of Arkansas for Medical Sciences (Protocol code number 3242; approved on 12/6/2011). Liver samples were obtained from male obese Zucker rats from a previous study (12). Rats (6 weeks old) were purchased from Harlan Laboratories (Indianapolis, IN) and following 1 week of acclimation, rats (*n* = 8–9 rats) were randomly assigned to a casein (CAS) based or a soy protein isolate (SPI) based diet. Both diets (CAS and SPI) were prepared by Harlan Teklad, Madison, WI, USA) with dietary protein, either casein (CAS, control) or soy protein isolate (SPI) with high isoflavones [3.24 mg total isoflavones/g protein (1.88 aglycone equivalents/g protein), Lot no. M330024462; Solae LLC, St. Louis, MO, USA]. The compositions of the two diets are described in Hakkak et al., 2011 (14). The rats were kept in individual cages and provided the CAS or SPI diet *ad libitum* for 16 weeks. After the rats were humanely killed, liver samples were obtained and flash frozen in liquid nitrogen and stored at −80°C.

### Transcriptomic Analysis

A phenol-chloroform solution was used to extract RNA from liver samples. A 1% agarose gel was used to determine RNA quality and concentrations were assessed with Take three micro volume plate with a Synergy HT multi-mode microplate reader (BioTek, Winooski, VT). RNA sequencing (RNAseq) of RNA samples was conducted using an Illumina HiSeq 100 base paired end read at the Research Support Facility (Michigan State University, East Lansing, MI). The CLC Genomics Workbench 8 software that incorporates the pipeline was used to map the base pair reads to *Rattus* genome assembly (version 4) (15). The read per million (RPM) of raw read data was transformed using log2 to stabilize the variance and by quantile normalization. Over 1,200 transcripts were differentially expressed (>1.3-fold difference and *P* < 0.05). Ingenuity Pathway Analysis (IPA) commercial software (Qiagen, CA) was for interpretation of the dataset. All RNAseq data are available in GEO (accession number GSE158553).

### Real Time Quantitative PCR (RT-qPCR)

Targeted gene expression was conducted using RT-qPCR to validate transcriptomic results. Briefly, RNA was extracted from samples using Trizol reagent (#15596018, Life Technologies) following the manufacture's recommendations, treated with DNAase, and reverse transcribed with qScript cDNA SuperMix (catalog #95048-100, Quanta Biosciences). Next, the cDNA (RT products) were amplified by RT-qPCR (Applied Biosystems 7,500 Real-Time PCR system) using the Power SYBR green Master Mix (catalog #4312074, Life Technologies). Primers including the 18S ribosomal housekeeping gene are shown in Table 1. Cycling conditions for the RT-qPCR were: 50°C for 2 min, 95°C for 10 min, followed by 40 cycles of a two-step amplification program with 95°C for 15 s and 58°C for 1 min. To exclude contamination with non-specific PCR products, we incorporated melting curve analysis by applying the dissociation protocol from the Sequence Detection system. The 2^−ΔΔCt^ method was used to establish the relative expressions of target genes in this study (16). Relative mRNA expression was obtained by normalizing CAS expression values to 1.0 for comparison with the SPI group.

**Table 1:** Oligonucleotide PCR primers based on the *Rattus norvegicus* genome.

**Gene**	**Accession No**.	**Primer sequence**	**Orientation**	**Product size (bp)**
NPTX2	NM_001034199.1	TCCGGGCACAAGAGATCATC	Forward	59
		GATGTTTCCAGGCATGTTCGT	Reverse	
Resp18	NM_019278.1	GCAGCGACATAAATGCCCAC	Forward	136
		CAGAACATGCCTTGGGGTACA	Reverse	
RGN	NM_031546.1	AGCGAGTTGGTGTAGATGCC	Forward	83
		GAACTTGGTTCCAATGGTGGC	Reverse	
SULT2A1	NM_131903.1	GAGCTGGATTTGGTCCTCAAGT	Forward	134
		CAGTCCCCAATAGTGCCTTTCC	Reverse	
PRSS32	NM_001106983.1	CACAAATCAACCGCTCCCAC	Forward	127
		TTCGAGAATGACCTGCTCCG	Reverse	
AMDHD1	NM_001191781.1	GTGGGCACTGATGGGCTTAT	Forward	123
		CACCAAACCTGGCAAGATGC	Reverse	
IL33	NM_001014166.1	CAGAATCTTGTGCCCTGAGC	Forward	124
		CGGAGTAGCACCTTATCTTTTTCT	Reverse	
Cidea	NM_001170467.1	AGGCCTTGTTAAGGAGTCTGC	Forward	84
		CATAAGCGCCCGCATAAACC	Reverse	
PRSS8	NM_138836.1	CCTACAATGGCGTCCACGTT	Forward	59
		TGACACCACCCATTGATTTGA	Reverse	
Gnai1	NM_013145.1	TGCAAGCCTGCTTCAACAGA	Forward	70
		AAGTCATTCAGGTAGTACGCCG	Reverse	
HIVEP2	NM_024137.1	TACACTCTGGCTGCTATGCAC	Forward	93
		GGGTGCATCAGGTTTCATCTGT	Reverse	
Magee1	NM_001079891.1	CCCACCTGGAGTGCATCTTT	Forward	134
		GCCCATCTTTGGCCCATTTG	Reverse	
Sdr16c6	NM_001109356.1	GCCATCTCTCACTTCTGGATTTG	Forward	101
		CCAACGACTCCTGCTATGCT	Reverse	
18S	NR_046237.1	AGTCCCTGCCCTTTGTACACA	Forward	60
		GCCTCACTAAACCATCCAATCG	Reverse	

### Statistical Evaluation

The analysis of qPCR data and regression analysis between qPCR and transcriptomic data (RNAseq) was assessed by Student's *t*-test with Graph Pad Prism version 6.00 for Windows (La Jolla, CA, USA) and differences were considered significant at *P* < 0.05.

Upstream regulator analysis by Ingenuity Pathway Analysis (IPA, Qiagen, CA) is based on a combination of; (a) the number and degree of differential expression of downstream target molecules in the existing dataset, and (b) on expected effects between transcriptional regulators and their target genes from published literature citations that have been curated and stored in the IPA program. Upstream regulator analysis determines the number of known targets or regulators within the user's dataset and compares each differentially expressed molecule to the reported relationship in the literature. If the observed expression is mostly consistent with either activation or inhibition of the transcriptional regulator a prediction is made and an activation z score is generated that is also based on literature-derived regulation direction (i.e., “activating” or “inhibiting”). Activation z scores >2.0 indicate that a molecule is activated whereas activation z scores of <-2.0 indicate that a target molecule is inhibited. The *p*-value of overlap, which determines if there is a statistically significant overlap between the dataset molecules and those regulated by an upstream regulator, is calculated using Fisher's Exact Test and the significance is attributed to *p*-values < 0.05.

## Results

### Top Differentially Expressed Genes: RT-qPCR and RNAseq Data

**Figure 1:**
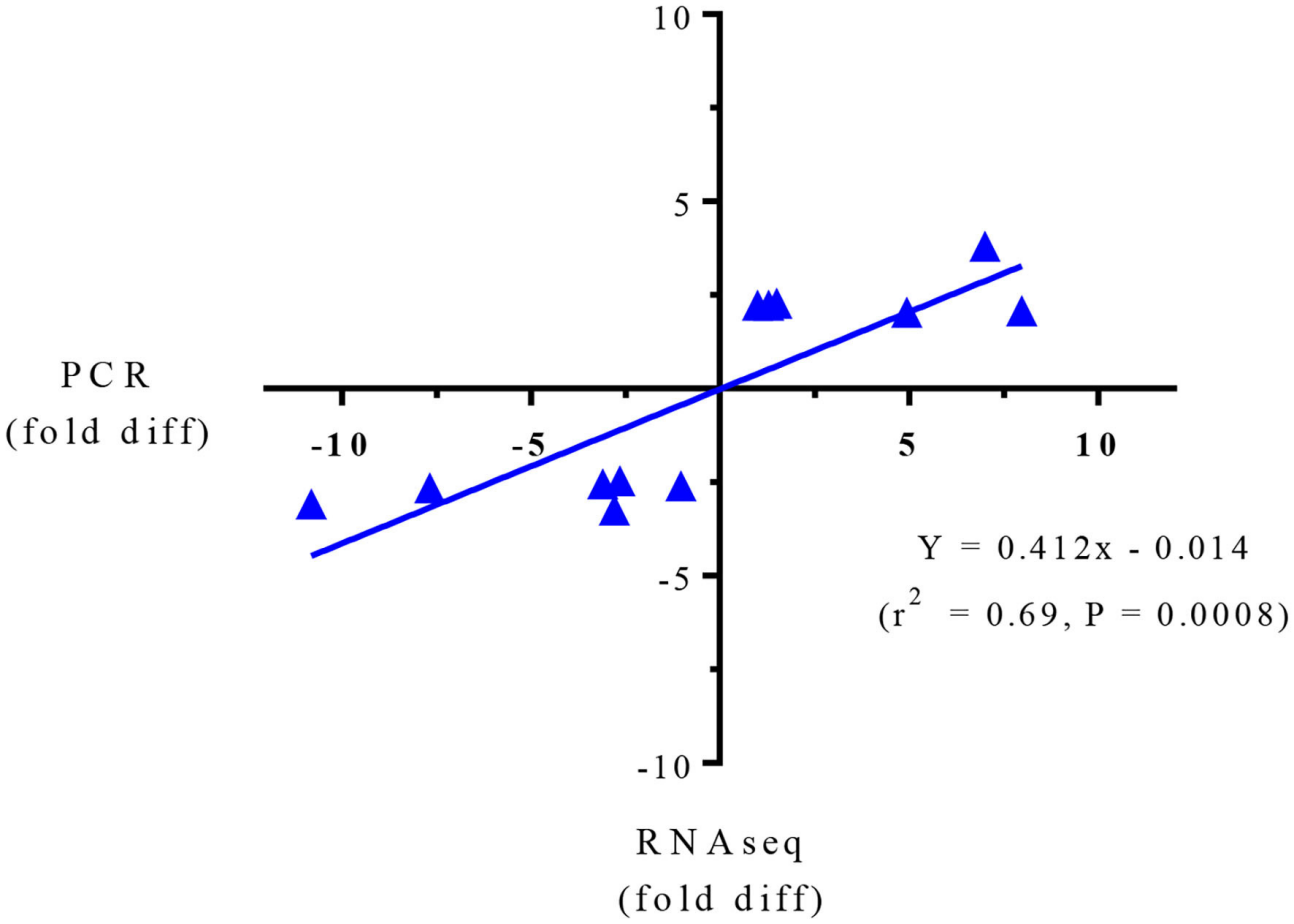
Regression analysis of RNAseq and PCR for targeted gene expression shown in Table 2.

Targeted gene expression was conducted by RT-qPCR on 12 of the most differentially expressed genes informed by the IPA program in the RNAseq dataset included NPTX2, PRSS32, Resp18, RGN, SULT2A1, AMDHD1 that were up-regulated, and Gnai, PRSS8. Cidea, MAGEE1, Sdr16c6 and HIVEP2 that were down-regulated in the SPI-fed compared to CAS-fed rats. Fold differences in mRNA expression by RNAseq and RT-PCR for these up- and down-regulated genes in the SPI-fed compared to CAS-fed rats are presented in Table 2. Regression analysis of mean values shown in Figure 1 indicate that there was a significant correlation (*r*^2^ = 0.69, *P* = 0.0008) between targeted PCR and RNAseq gene expression. A more detailed discussion on these targeted genes and their probable involvement in the development and progression of NAFLD is provided in Kozaczek (17).

**Table 2:** Comparison of mean mRNA expression (fold difference) in liver of obese rats fed soy protein isolate (SPI) or casein (CAS) diets for 16 weeks obtained by RNAseq and RT-PCR^a^.

**Gene symbol**	**Gene name**	**RNAseq (Fold diff)**	**PCR (Fold diff)**
NPTX2	Neuronal pentraxin 2	3.80	7.01
PRSS32	Protease, Serine 32	2.28	1.50
Resp18	Regulated Endocrine Specific Protein 18	2.22	1.00
RGN	Regucalcin	2.22	1.29
SULT2A1	Sulfotransferase Family 2A Member 1	2.07	7.98
AMDHD1	Amidohydrolase Domain Containing 1	2.04	4.94
Gnai	G Protein Subunit Alpha I1	−3.24	−2.79
PRSS8	Protease, Serine 8	−3.09	−10.81
Cidea	Cell Death Inducing DFFA Like Effector A	−2.65	−7.68
MAGEE1	MAGE Family Member E1	−2.60	−1.03
Sdr16c6	Short Chain Dehydrogenase/Reductase Family 16C Member 6	−2.55	−3.10
HIVEP2	Human Immunodeficiency Virus Type I Enhancer Binding Protein 2	−2.46	−2.65

*These mean values were used in correlation analysis (see Figure 1)*.

a *Values represent mean of n = 8. Positive and negative values indicate up- and down-regulation of gene expression, respectively, in livers of obese rats fed soy protein isolate-based vs. casein-based diets*.

### Upstream Regulators

Upstream regulator predictions in the RNAseq data provide insight into potential mechanisms in this study by which SPI feeding ameliorates liver steatosis in NAFLD. A partial list of upstream regulators predicted to be activated or inhibited in SPI-fed rats vs. CAS-fed obese rats after 16 weeks of feeding is presented in Table 3. The top upstream regulators predicted to be inhibited in SPI-fed rats include tumor necrosis factor (TNF), interferon gamma (IFNG), colony stimulating factor 2 (CSF2), resistin-like beta (RETNLB) and TNF receptor 1 beta (TNFRSF1B) (Table 3A). Top upstream regulator genes predicted to be activated in SPI-fed rats include Acyl-CoA (ACOX1), hepatocyte nuclear factor 4 alpha (HNF4A), insulin induced gene 1 (INSIG1), hepatocyte nuclear factor 1 alpha (HNF1A), immunoglobulin G (IgG), and Aryl hydrocarbon receptor (AHR). There were 32 genes predicted to be inhibited in SPI-fed rats and 15 genes that were predicted to be activated in the SPI-fed rats (17). Each upstream regulator prediction point toward fundamental mechanisms that are involved in attenuation of liver steatosis provided by feeding the SPI to the obese rats for a 16 week period.

**Table 3:** A partial list of upstream regulators that were predicted to be inhibited (A) or activated (B) in liver of rats provided a diet with soy protein isolate (SPI) compared to those consuming a Casein-based diet for 16 weeks.

**Upstream regulator**	**Name**	**Molecule type**	**Activation *z*-score**
**A**			
TNF	Tumor necrosis factor	Cytokine	−3.37
IFNG	Interferon Gamma	Cytokine	−3.30
CSF2	Colony Stimulating Factor 2	Cytokine	−2.29
RETNLB	Restin like beta	Other	−2.14
TNFRSF1B	Tumor necrosis factor receptor 1 beta	transmembrane receptor	−2.00
**B**			
ACOX1	Acyl-CoA oxidase 1	Enzyme	4.04
HNF4A	Hepatocyte nuclear factor 4 alpha	transcription regulator	3.44
INSIG1	Insulin induced gene 1	Other	2.71
HNF1A	Hepatocyte nuclear factor 1 homeobox A	transcription regulator	2.55
IgG	Immunoglobulin G	Complex	2.17
AHR	Aryl hydrocarbon receptor	ligand-dependent nuclear receptor	2.15

*Predicted inhibition (blue); Predicted activition (orange)*.

**Figure 2:**
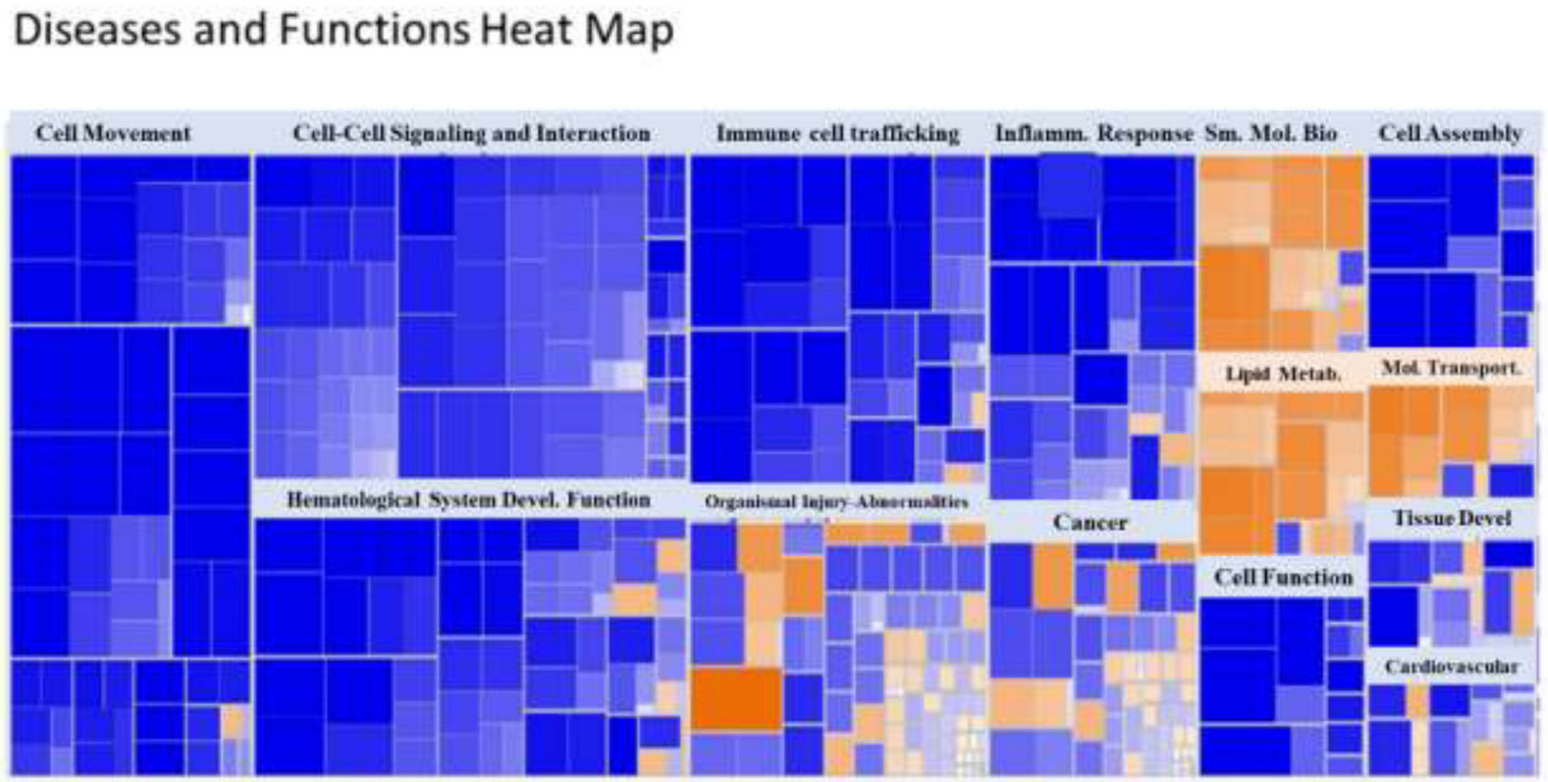
Heat map of diseases and functions arranged by Z-score with the IPA program. Functions in blue indicate inhibition while those in orange indicate activation in SPI vs. CAS-fed rats. Darker colors indicate greater activation or inhibition of function.

**Figure 3:**
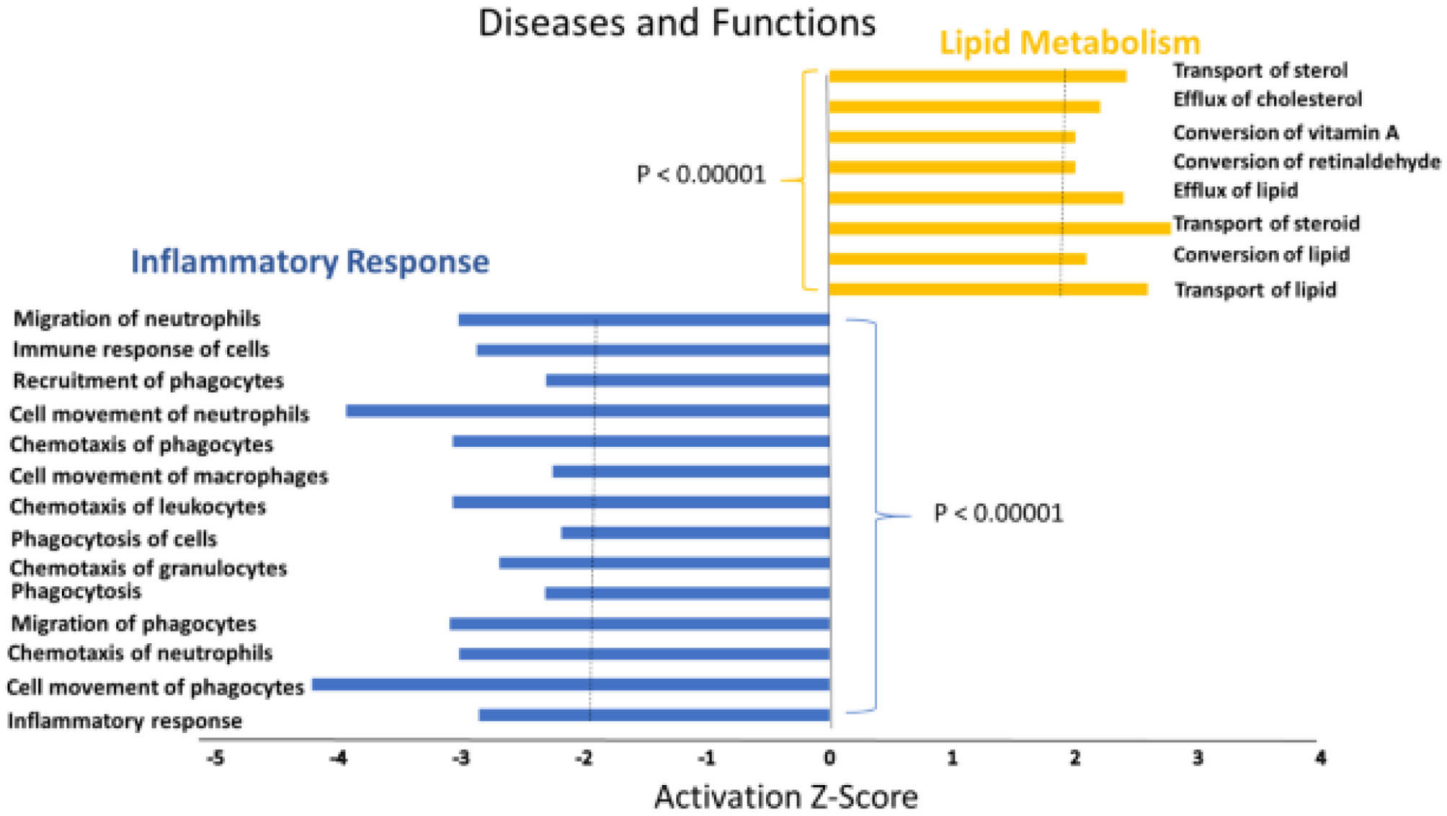
Specific functions predicted to be activated associated with lipid metabolism (orange) or predicted to be inhibited (blue) associated with inflammation in the liver of obese Zucker rats provided diets containing SPI-based diet compared to those provided a CAS-based diet for 16 weeks.

A heat map of the RNAseq data generated by IPA software is presented in Figure 2. Blue represents diseases and functions that were predicted to be inhibited whereas orange represents those predicted to be activated in the SPI-fed vs. CAS-fed rats. The darker the color the stronger to prediction based on activation Z-scores calculated by the IPA program. Specific functions predicted to be activated or inhibited in the dataset (with activation Z-scores of < −2.0 or >2.0, respectively) within two broad diseases and functions classification (lipid metabolism and inflammatory response) are presented in Figure 3. Each of the specific processes listed with lipid metabolism are predicted to be enhanced in the SPI-fed rats whereas each of the processes listed under inflammatory response were predicted to be inhibited in the SPI-fed rats. The list of functions under lipid metabolism involving lipid transport or efflux is consistent with the observation of reduced liver steatosis in the SPI- compared to CAS-fed rats. Furthermore, the list provided under inflammatory response clearly indicates that inflammation would be inhibited in the SPI-fed rats.

**Figure 4:**
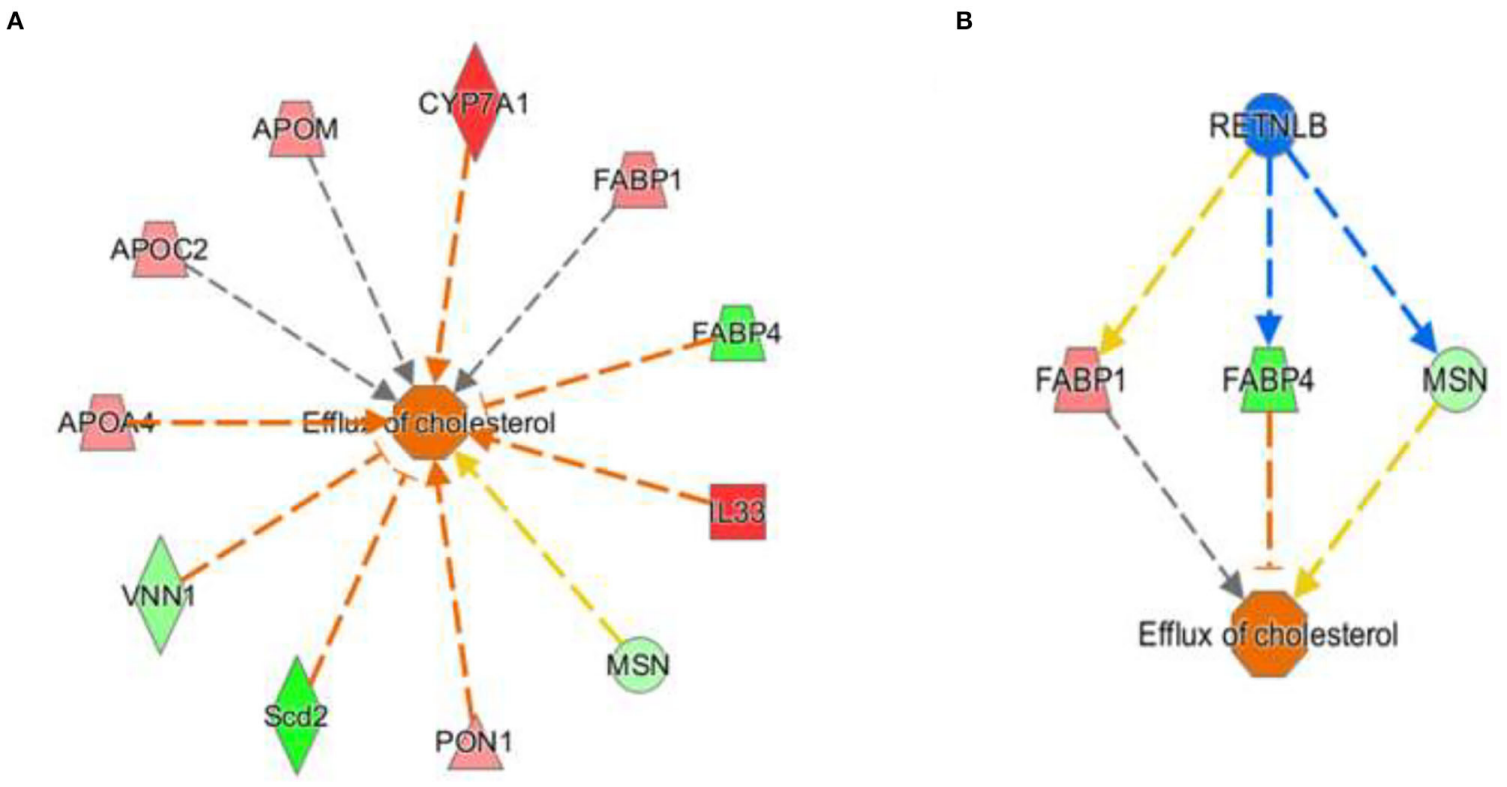
Gene expression networks leading to prediction of enhanced efflux of cholesterol in the RNAseq data in liver of 16 weeks old obese rats fed CAS- and SPI-based diets. **(A)** Network of gene expression in the dataset used to calculate the activation *Z* score of efflux of cholesterol. Genes in pink or red were upregulated in the SPI-fed rats whereas those in green were down-regulated in SPI- vs. CAS-fed rats. Genes and their individual fold difference expression values are; APOA4 (apolipoprotein A4, 1.37), APOC2 (apolipoprotein C2, 1.36), APOM (apolipoprotein M, 1.38), cytochrome P450 family 7 subfamily A, member 1,1.98), FABP1 (fatty acid binding protein 1, 1.41, FABP4 (fatty acid binding protein 4, −1.91), IL33, (Interleukin 33, 2.32), MSN (moesin,-1.32), PON1 (paraoxanase 1, 1.35), Scd2 (stearoyl-Coenzyme A desaturase 2, −2.42), VNN1, (vanin 1, −1.46). **(B)** This is a regulatory network that predicts that RETNLB (restin like protein beta) transcription factor would be predicted to be inhibited in the SPI-fed rats.

**Figure 5:**
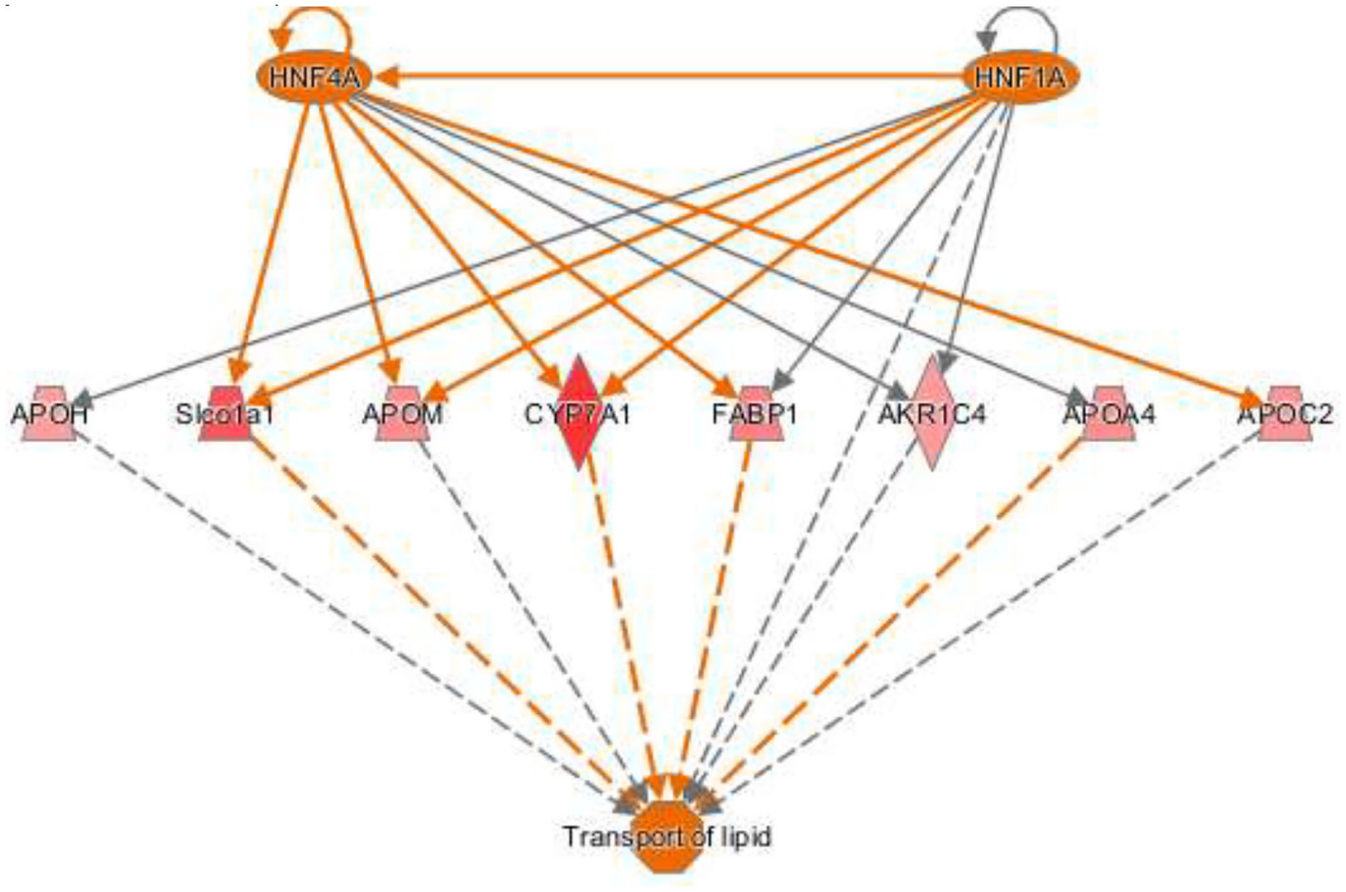
Regulator network for transport of lipid with two upstream regulators. The hepatocyte nuclear factors one (HNF1) homeobox A (HNF1A) and HNF1 factor 4 alpha (HNF4A) are predicted to be activated and enhance lipid transport in SPI-fed rats through the network of genes shown in the figure that include: APOH (apolipoprotein H, 1.32), Slco1a1 (solute carrier organic ion transporter family member 1A1, 1.62), APOM (apolipoprotein M, 1.38), CYP7A1 (cytochrome P450 family 7 subfamily A member 1), FABP1 (fatty acid binding protein 1, 1.42), AKR1C4 (also-keto reductase family member c14, 1.45), APOA4 (apolipoprotein A4, 1.37), and APOC2 (apolipoprotein C2, 1.36).

**Figure 6:**
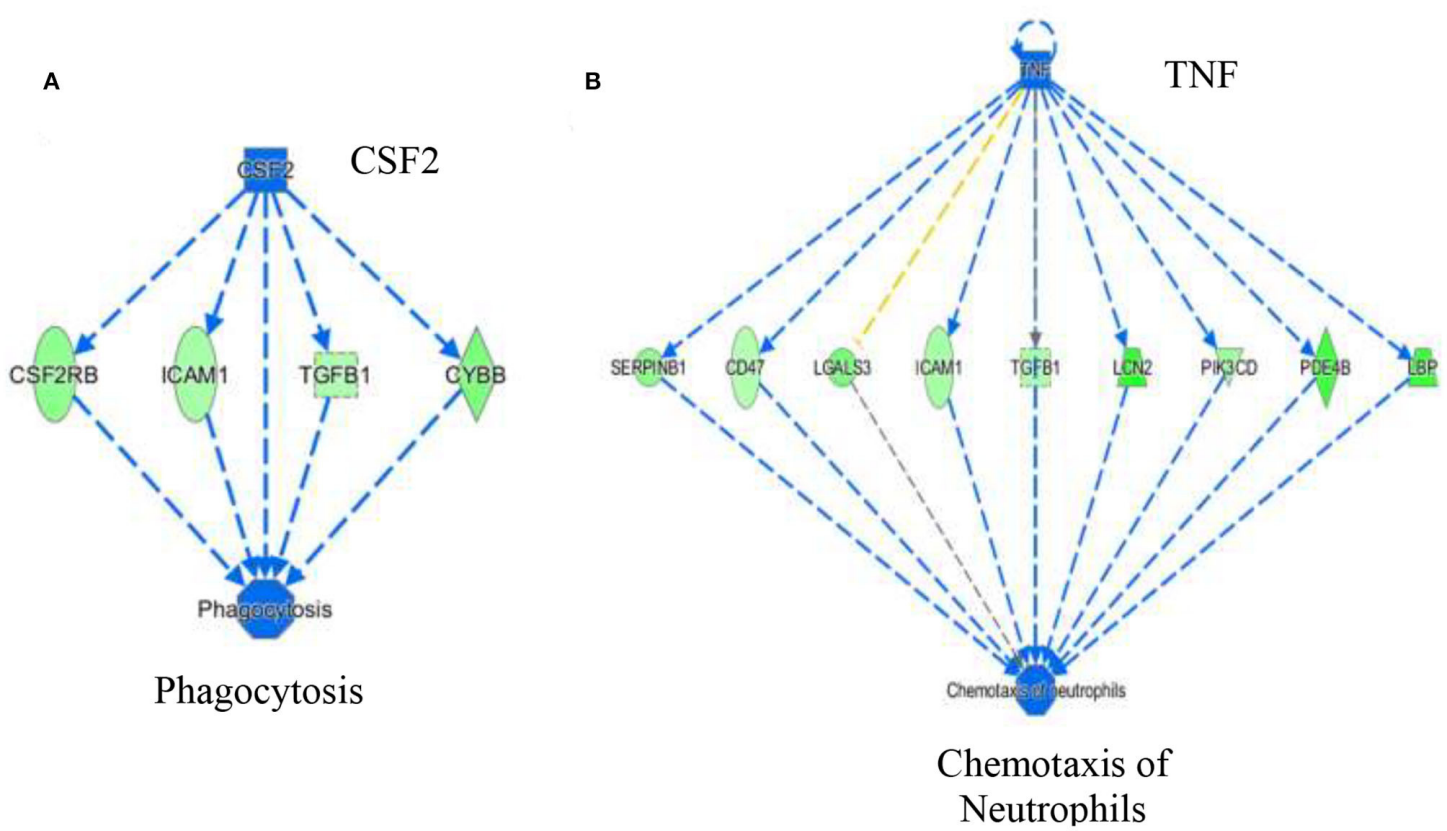
Regulator networks associated with specific components of inflammatory response of phagocytosis **(A)** and chemotaxis of neutrophils **(B)** that were predicted to be inhibited in SPI-fed compared to CAS-fed rats. In **(A)** the transcription factor colony stimulating factor 2 (CSF2) was predicted to be in inhibited in SPI-fed rats based on downregulation of CSF2RB, ICAM1, TGFB1 and CYBB. In **(B)**, TNF (tumor necrosis factor) was predicted to be inhibited in SPI-fed rats based on the expression of genes shown in the network. Genes and fold differential expression in the regulatory networks are: CSFR2B (colony stimulating factor 2 receptor 2 beta common subunit, −1.47), ICAM1 (intracellular adhesion molecule 1, −1.36), TGFB1 (transforming growth factor beta 1, −1.33), CYBB (cytochrome b-245 beta chain, −1.57), SERPINB1 (serpin family B member 1, −1.50), CD47 (CD47 molecule, −1.30), LGALS3 (galectin 3, −1.62), LCN2 (lipocalin 2,−2.10), PIK3CD (phosphoinositol-4,5 bisphosphate 3 kinase catalytic subunit D, −1.41), PDE4B (phosphodiesterase 4B, −1.93), and LBP (lipopolysaccharide binding protein, −1.99).

The activation *Z*-scores of processes presented in Figure 3 were calculated by the IPA program and based on the differential expression of genes in the dataset and on the relationships of gene expression with the function reported in the scientific literature. An example of one of the networks generated by the IPA program analysis of the dataset for efflux of cholesterol is presented in Figure 4A. The IPA program can also provide predictions of activation or inhibition of upstream transcription factors in regulator networks that connect transcription factors to specific diseases or function through differentially expressed molecules in the functional network. In the example shown in Figure 4B, the transcription factor RETNLB was predicted to be inhibited in SPI- compared to CAS-fed rats. Additional examples of regulator networks are presented in Figures 5, 6. In Figure 5, HNF1A and HNF4A are transcription factors predicted to be activated in the SPI-fed rats that would contribute to lipid transport from the liver. Two regulator networks indicating that phagocytosis and chemotaxis of neutrophils are predicted to be inhibited in SPI-fed rats through down-regulation (inhibition) of CSF2 and TNF are shown in Figure 6.

## Discussion

NAFLD is characterized by an unhealthy accumulation of lipids within hepatocytes accompanied by inflammation of the liver tissue. Here we demonstrate that long-term feeding of SPI enhances lipid metabolism and decreases several components of inflammation response (Figure 3). Several functions that were predicted to be activated (e.g., lipid transport, efflux, and conversion of lipid) in SPI-fed rats are processes that could reduce lipid accumulation in hepatocytes. Similarly, processes and functions involved in inflammatory response such as phagocytosis, chemotaxis of neutrophils, and immune response of cells, were inhibited in SPI-fed rats, thus ameliorating NAFLD symptoms. A wide range of molecules were predicted to be activated or inhibited in liver of obese rats fed an SPI- compared to CAS-based diet. As discussed below, these predictions point toward mechanisms by which SPI ameliorates liver steatosis and can serve as hypotheses to be tested in future studies.

Acyl-CoA Oxidase 1 (ACOX1) (Table 3), also called Peroxisomal acyl-coenzyme A oxidase 1, is a transcription factor predicted to be activated (*z* score = 4.04) in liver of rats provided a SPI-based diet. Unlike most medium to long chain fatty acids whose oxidation takes place in mitochondria, very long fatty acids are degraded in peroxisomes (18). ACOX1 catalyzes the initial step of the peroxisomal beta-oxidation of very long fatty acids in the fatty acid degradation pathway (19). A study with a point mutation of ACOX1 showed that beta-oxidation provides a clear link between fat metabolism and immune responsiveness (20). In this study, the authors found an increase in the hepatic neutrophil infiltration when ACOX1 was not functioning properly. This finding is consistent with the results in our study predicting decreased hepatic migration and chemotaxis of neutrophils (Figures 3, 6B) when ACOX1 is activated in SPI feeding. In addition, a recent study concluded that ACOX1 is the post-transcriptional target of miR-222 (21), a microRNA already being investigated in relation to NASH, cancer, and inflammatory diseases (22). However, ACOX1 is not as strongly related to NAFLD as it is with NASH and hepatic cell carcinoma (23). Wang et al. (21) reported that miR-222 inhibits ACOX1 and accumulation of triglycerides is promoted. These results concur with findings in the present study in which ACOX1 is predicted to be highly activated in SPI diet; thus, preventing accumulation of triglycerides and inflammation. Since many microRNAs are linked to the pathology of insulin resistance, NAFLD, and fibrosis (24) it would be reasonable to link a decreased expression or inhibition of miR-222 by the consumption of a diet high in soy proteins, although the mechanisms remain unknown. Further research it's needed.

Both HNF4A and 1 HNF1A (Table 3B) were predicted to be strongly activated in this study (z score = 3.44 and 2.55 respectively) in the liver of SPI-fed rats. HNF4A is a nuclear transcription factor known to regulate several hepatic genes including the related transcription factor HNF1A (Figure 5) and other genes related to lipid metabolism (25). There is evidence supporting HNF4A as a universal transcription regulator of hepatic cytochrome P450 (CYP) genes (26). HNF1A is mainly expressed in liver but is also expressed in the pancreas and kidney (27). HNF4A and HNF1A are major promoters of hepatic differentiation and maturation (28, 29). With its involvement in lipid metabolism, HNF4A has been demonstrated to be crucial in the gene network of NASH connected to metabolic diseases (30). Mutations in this gene have also been linked with both diabetes type 2 and maturity onset diabetes of the young (MODY), a term that groups a set of hereditary diabetes mellitus (31). An amplification of this gene has been found to be associated with colorectal cancer (32). In the early 2000's, Lazarevich et al. (33) not only found a strong correlation between the downregulation or lack of function of HNF4A and the progression of hepatocellular carcinoma (HCC), but also that restoration of HNF4A expression could reverse the HCC phenotype in a mouse model. Ning et al. (34) established that the increased expression of HNF4A inhibits HCC and alleviates hepatic fibrosis probably through the repression of beta-catenin signaling pathway, and proposed the administration of HNF4A as a possible future pathological treatment. The search for biomarkers HCC derived from NAFLD has shown the same trend (35): an imbalance of important transcription factors including HNF4A. Our study predicts HNF4A to be highly activated in the SPI-fed rats, along with the upregulation of several CYPs and apolipoproteins (Figure 5), which could be exerting a protective influence against lipid accumulation in the liver tissue; thus, preventing inflammation and the progression to NAFLD in the long-term.

Several molecules associated with inflammatory response were predicted to be inhibited in SPI-fed rats compared with the control. These will be discussed individually below.

Tumor necrosis factor (TNF), a pro-inflammatory cytokine secreted by various cells, is released in response to inflammatory stimuli (36). TNF was predicted to be inhibited (z score = −3.37) in the livers of SPI-fed rats (Table 3, Figure 6B). This protein also promotes apoptosis and necrosis, and increases in response to endotoxins and bacterial lipopolysaccharide (LPS) (37, 38). Lipopolysaccharide binding protein (LBP) was down-regulated in SPI-fed rats and was one of the downstream molecules used by IPA in predicting TNF inhibition (Figure 6B). There are many ways that TNF could contribute to NAFLD and NASH pathophysiology. TNF is a key promoter of other inflammatory cytokines and molecules related with hepatic steatosis and fibrosis (39). It has been suggested that TNF increases hepatic fat deposition via activation or upregulation of sterol regulatory element binding protein-1c (SREBP-1c) that in turn controls fatty acid synthase (FAS) (40). TNF has the ability to lower insulin receptor expression that contributes to the development of insulin resistance (41). Furthermore, inhibition of TNF in NASH patients has been reported to be beneficial either in ameliorating or reversing NASH pathology (42). In a 4 year study, TNF serum levels were reported to be increased in patients with NAFLD, and are associated with the stage of the disease in concordance with the literature (43). Our study predicted a strong inhibition of TNF under a SPI diet (Figure 6B) and neutrophil chemotaxis was broadly predicted to be inhibited. All the genes involved in this network were downregulated in this study (see Figure 6B for a full list of the genes). Conversely, in the CAS-fed rats, TNF might promote the chemotaxis of neutrophils in the liver by directly participating in the upregulation of the same genes. Nevertheless, the underlying mechanisms are not completely understood.

Colony stimulating factor 2 (CSF2) (Table 3A and Figure 6A) or granulocyte-macrophage colony-stimulating factor (GM-CSF) was predicted to be inhibited in SPI-fed rats at 16 weeks (activation *z* score = −2.29). Its receptor, CSF2 receptor beta (CSF2RB) was found to be down-regulated at 8 weeks on the SPI diet (13). CSF2 is a cytokine involved in inflammatory response that modulates the production and differentiation of macrophages and granulocytes (44, 45). Morrison et al. (46) found a correlation of the up-regulation of CSF2 in humans and Ldlr−/−. Leiden mice with NASH. Thus, our study concurs with Morrison et al. (46) since CSF2 was predicted to be inhibited in the liver of rats fed with the SPI diet (Figure 6A) in a pathological state previous to NASH. Conversely, our study predicted CSF2 to be highly activated in CAS-fed rats, which presented inflammation in liver tissue, and directly promoted phagocytosis through the activation of important target molecules (see Figure 6A).

## Conclusions

The results of the analysis conducted in this study provide a clear indication that long-term SPI feeding attenuates liver steatosis by enhancing fat metabolism and lipid transport from the liver while simultaneously lowering activity of upstream regulator genes that would inhibit inflammation. Each of the predictions of activation or inhibition of genes that were calculated from expression of downstream molecules and literature citations of similar or dissimilar relationships should be considered as hypotheses to be tested in future studies.

## Data Availability Statement

The datasets presented in this study can be found in online repositories. The names of the repository/repositories and accession number(s) can be found below: NCBI Gene Expression Omnibus (https://www.ncbi.nlm.nih.gov/geo/query/acc.cgi?acc=GSE158553).

## Ethics Statement

The animal study was reviewed and approved by IACUC at the University of Arkansas for Medical Sciences (protocol no. 3242 approved 12-6-11).

## Author Contributions

RH: responsible for experimental design, diets, and animals. BK: bioinformatics. MK and DA: targeted mRNA analysis. MK, BK, KL, WB, and RH: data analysis and interpretation. All authors approved the final version of the manuscript.

## Conflict of Interest

The authors declare that the research was conducted in the absence of any commercial or financial relationships that could be construed as a potential conflict of interest.
